# Effect of Endoscopic Retrograde Appendicitis Therapy on Postoperative Gastrointestinal Function in Patients With Chronic Fecalith Appendicitis

**DOI:** 10.1155/grp/4306502

**Published:** 2026-06-26

**Authors:** Yao Wu, Peichen Xia, Jing Chen, Yao Liu, Yan Yang, Yanyan Wang, Wenzhuo Jiang, Yu Fan, Hong Zhu, Xiaomeng Jiang

**Affiliations:** ^1^ Department of Gastroenterology, Sir Run Run Hospital, Nanjing Medical University, Nanjing, Jiangsu, China, njmu.edu.cn; ^2^ Department of Medical Laboratory Technology, Kangda College of Nanjing Medical University, Lianyungang, Jiangsu, China, njmu.edu.cn; ^3^ Cancer Institute, the Affiliated People′s Hospital, Jiangsu University, Zhenjiang, Jiangsu, China, ujs.edu.cn; ^4^ Department of Gastroenterology, Jiangsu Province Hospital, The First Affiliated Hospital of Nanjing Medical University, Nanjing, Jiangsu, China, njmu.edu.cn

**Keywords:** chronic fecalith appendicitis, ERAT, gastroenteric function, GSRS, LA

## Abstract

**Background:**

This study compared postoperative gastrointestinal function recovery between endoscopic retrograde appendicitis therapy (ERAT) and laparoscopic appendectomy (LA) in patients diagnosed with chronic fecalith appendicitis.

**Methods:**

This was a retrospective study enrolling patients with chronic fecalith appendicitis, who were divided into the ERAT group and the LA group. Gastrointestinal manifestations were recorded, and the Gastrointestinal Symptom Rating Scale (GSRS) score was applied to evaluate gastrointestinal function. Preoperative and postoperative symptomatic improvement and GSRS scores were compared across groups.

**Result:**

A total of 101 eligible patients were recruited and divided into the ERAT group (*n* = 40, 39.60%) and the LA group (*n* = 61, 60.40%). All ERAT procedures were completed successfully, with 100% rates of successful intubation and fecalith removal. No severe adverse events were reported postoperatively in either cohort. Compared with LA, ERAT presented significantly shorter operative duration and postoperative hospital stay (statistically significant differences). For GSRS score assessment, the median preoperative, postoperative scores and score reduction were 5 (4), 1 (1.75), and 3.5 (1) in the ERAT group, versus 4 (2), 3 (3), and 1 (2) in the LA group. The ERAT group exhibited a significantly larger magnitude of symptom improvement (*p* < 0.001, *z* = −5.484). Multivariate logistic regression analysis indicated that patients undergoing ERAT obtained a significantly higher degree of GSRS score improvement (*p* < 0.001, odds ratio = 2.137, 95% confidence interval: 1.461–3.126). Analysis of individual GSRS domains demonstrated that improvements in abdominal pain, dyspepsia and diarrhea were significantly more prominent in the ERAT group.

**Conclusion:**

As an organ‐preserving endoscopic procedure, ERAT retains appendiceal function and protects gastrointestinal physiological function. Our findings offer meaningful clinical references of treatment strategies for chronic fecalith appendicitis.

## 1. Introduction

The overall incidence of appendicitis worldwide is about 0.1% [1], and the lifetime risk of an individual developing appendicitis is as high as 7%–8% [2]. Obstruction of the appendix cavity is the main cause of chronic appendicitis. Appendicular fecalith is formed by mineral precipitation and hard stool, which often leads to obstruction of the appendix cavity, causing appendicular ischemia, inflammation, and even gangrene, perforation, and widespread peritonitis. Studies have shown that the risk of complications in fecalith appendicitis is significantly higher than that in those without fecalith [3]. Clinically, laparoscopic appendectomy (LA) has historically been the primary intervention for appendicitis. Nonetheless, resecting the appendix—a vital immune organ and natural reservoir for intestinal flora—is associated with multiple adverse events. Of note, the rate of negative appendectomy continues to range from 8% to 15% in routine clinical work [4]. At the same time, studies have shown that the incidence of complications after appendectomy is as high as 20.5% [5]. Common complications include incisional infection, abdominal infection, small intestinal obstruction, incisional hernia, and other complications such as diarrhea and cardiovascular accidents [6]. These findings imply that LA could exert adverse effects on gastrointestinal function.

In 1995, Said et al. [7] applied the principles and techniques of endoscopic retrograde cholangiopancreatography (ERCP) to manage patients with atypical acute appendicitis. In 2002, Liu et al. [8] provided a detailed description of the endoscopic retrograde appendicitis therapy (ERAT) procedure. This effective method for managing appendicitis has pioneered a new strategy for appendiceal preservation and functional maintenance. As of 2023, more than 10,000 ERAT operations have been completed successfully in China [9]. The procedure effectively relieves chronic recurrent abdominal pain, bloating, diarrhea, and constipation in affected patients [9]. Collectively, these outcomes demonstrate that ERAT can ameliorate gastrointestinal dysfunction and related abdominal symptoms due to chronic appendicitis.

Gastrointestinal Symptom Rating Scale (GSRS) is a well‐established tool for quantifying gastrointestinal symptoms, including abdominal pain, reflux, diarrhea, constipation, and indigestion [10]. Its reliability and effectiveness have been demonstrated in different populations, such as gastroesophageal reflux disease, irritable bowel syndrome, and depression [11–13]. However, there are no relevant studies on the improvement of gastrointestinal function and GSRS score after ERAT in patients with chronic fecalith appendicitis.

In this retrospective study, the GSRS was applied to compare gastrointestinal function outcomes after ERAT versus LA for chronic fecalith appendicitis. Our data provide a reference for individualized treatment planning.

## 2. Patients and Methods

### 2.1. Patients

Clinical data of patients with chronic fecalith appendicitis who were admitted to Sir Run Run Hospital Affiliated to Nanjing Medical University from January 2022 to January 2026 were retrospectively collected. The patients underwent either ERAT or LA. This study was approved and supervised by the Ethics Committee of the Sir Run Run Hospital Affiliated to Nanjing Medical University (Approval No. 2022‐SR‐003). Each patient and/or family member signed an informed consent prior to study participation and surgery.

Inclusion criteria are as follows: (1) Some patients had a history of acute appendicitis, whereas others did not. All subjects experienced recurrent right lower quadrant pain over a period of 3 months or longer, with a minimum of two symptomatic attacks; (2) localized tenderness in the right lower abdomen with or without rebound pain in the right lower abdomen; (3) abdominal computed tomography confirmed appendiceal fecaliths and typical chronic inflammatory signs (appendiceal wall thickening and luminal dilatation) while excluding alternative etiologies for chronic abdominal pain.

Exclusion criteria are as follows: (1) perforation of appendicitis, periappendiceal abscess, and appendiceal tumor; (2) previous medical history, abdominal CT and/or gastroenterological endoscope suggest other causes of abdominal pain, reflux, indigestion, constipation, diarrhea, and so on.; (3) contrast media or iodine allergy; and (4) pregnancy and severe abnormal cardiopulmonary function.

Prior to treatment, patients were thoroughly informed about ERAT, LA, and their possible complications. The final treatment strategy was decided in accordance with the inclusion criteria and individual patient wishes.

### 2.2. Clinical Data Collection and Follow‐Up

All baseline and clinical data were retrospectively extracted from electronic medical records at Sir Run Run Hospital Affiliated to Nanjing Medical University. Baseline characteristics comprised gender and age. Collected clinical data included white blood cell (WBC) count, C‐reactive protein (CRP), preoperative visual analogue scale (VAS) score, fecalith diameter, surgical procedure, ERAT intubation success rate, ERAT lithotomy success rate, operative time, length of hospital stay, postoperative complications and gastrointestinal symptoms. Assessed gastrointestinal symptoms covered abdominal pain, hunger pain, nausea, vomiting, acid regurgitation, heartburn, borborygmus, abdominal distension, belching, increased flatus, decreased defecation, increased defecation, hard stools, loose stools, tenesmus and incomplete evacuation, as well as the GSRS score [10]. The GSRS, developed by Dimenas, consists of 15 items divided into five domains: abdominal pain (two items), reflux (three items), dyspepsia (four items), constipation (three items), and diarrhea (three items). In this study, a simplified 4‐point Likert scale (0–3 points) was used: 0 = *no symptoms*, 1 = *mild symptoms*, 2 = *moderate symptoms*, and 3 = *severe symptoms*. The original version adopts a 7‐point Likert scoring system. In this study, a simplified 0–3 scoring scale was applied to reduce the respondents′ burden. The Chinese version of GSRS has been validated [14]. Higher scores indicate more severe symptoms. All patients were followed up by outpatient service or telephone. All patients were followed up via outpatient visits or telephone interviews at 3 months after treatment. Changes in clinical symptoms and GSRS scores were documented during follow‐up. All follow‐up assessments and GSRS evaluations were performed by one experienced researcher to ensure consistency.

### 2.3. Statistical Analysis

For statistical analysis, continuous variables with a normal distribution were expressed as mean ± standard deviation, and the two‐sample *t*‐test was used to compare differences between groups. Nonnormally distributed continuous variables were summarized using median and interquartile range (IQR), with the Wilcoxon rank‐sum test for intergroup comparison. Categorical variables were presented as proportions, and the chi‐square test was employed accordingly. Binary logistic regression was performed for multivariate analyses. Statistical significance was defined as *p* < 0.05. Data processing and analyses were conducted with SPSS Version 29.0 (IBM, Armonk, New York, United States).

## 3. Results

### 3.1. Univariate Analysis of Baseline Characteristics and Clinical Data (Table 1)

**Table 1:** Univariate analysis.

Characteristics	Total	ERAT	LA	*p*	*t*/*z*
Total	101	40 (39.60%)	61 (60.40%)		
Age (years)	41.06 (±16.18)	42.08 (±15.76)	40.49 (±16.54)	0.887	−0.509
Sex				0.572	—
Male	47 (46.53%)	20 (50%)	27 (44.26%)		
Female	54 (53.47%)	20 (50%)	34 (55.74%)		
WBC (10^9^/L)	5.35 (2.08)	5.35 (2.08)	5.4 (2.13)	0.898	−0.129
CRP (mg/L)	1.38 (2.2)	1.2 (2.2)	1.45 (2.2)	0.957	−0.054
VAS	4 (2.5)	4 (2.75)	5 (2.5)	0.850	−0.189
Diameter of fecalith (mm)	4.1 (1)	4.4 (1.23)	4.1 (1.3)	0.410	−0.823
Operative duration (min)	40 (18.5)	36 (10)	40 (20)	**0.035**	−2.114
Hospital stay (days)	3 (1)	3 (1)	4 (1)	**0.002**	−3.029
GSRS score (preoperation)	4 (3)	5 (4)	4 (2)	0.063	−1.861
GSRS score (postoperation)	2 (3)	1 (1.75)	3 (3)	**< 0.001**	−3.512
GSRS score (difference value)	2 (2.5)	3.5 (1)	1 (2)	**< 0.001**	−5.485

*Note:* The bolded values indicate that statistical significance was defined as *p* < 0.05

**Figure 1:**
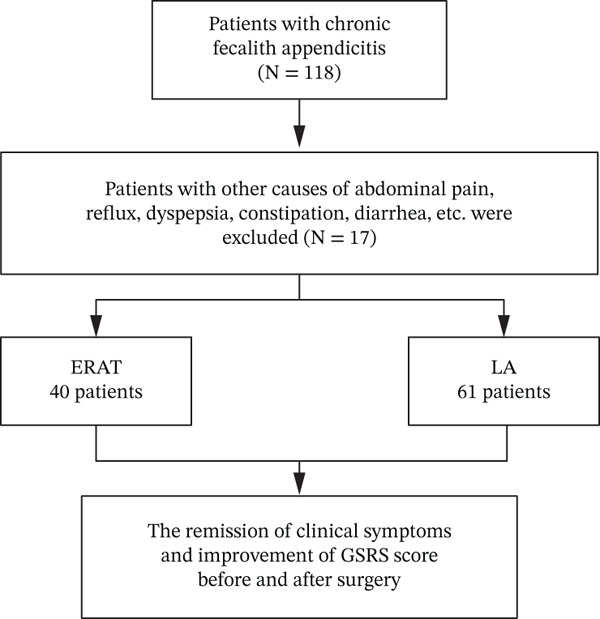
Flow chart.

**Figure 2:**
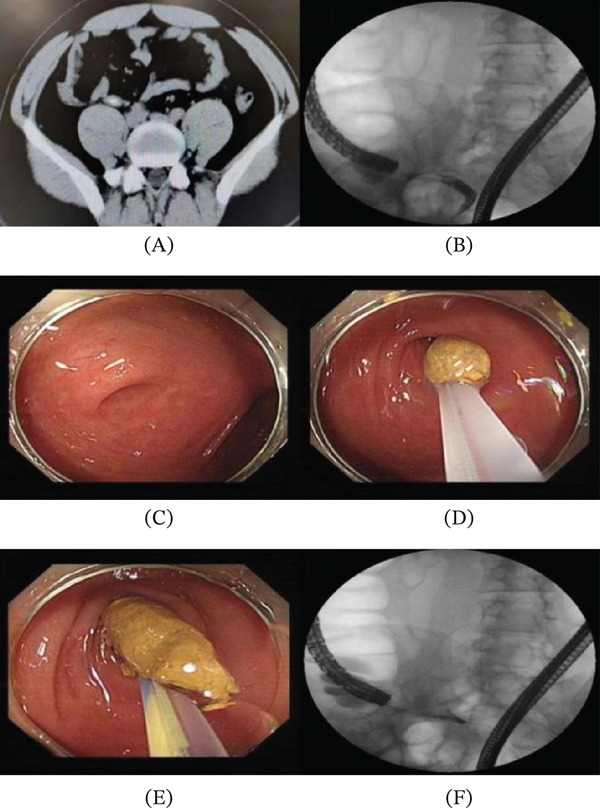
ERAT operation for chronic fecalith appendicitis. (A) Abdominal CT showed high‐density imaging in the appendiceal cavity; (B) X‐ray imaging revealed multiple filling defects in the appendix; (C) the colonoscopy indicated that the appendix opening was normal; (D + E) extraction of appendix fecalith; (F) X‐ray imaging showed no filling defect in the appendix.

A total of 101 patients were enrolled in this study, including 40 (39.60%) who received ERAT and 61 (60.40%) who underwent LA (Figure 1). The overall mean age was 41.06 ± 16.18 years. The mean age was 42.08 ± 15.76 years in the ERAT group and 40.49 ± 16.54 years in the LA group. Of all participants, 47 (46.53%) were male and 54 (53.47%) were female. The ERAT group consisted of 20 males and 20 females, whereas the LA group included 27 males and 34 females. No significant intergroup differences were found in age and gender. Likewise, WBC count, CRP level, preoperative VAS score, and fecalith diameter were comparable between the two groups, indicating good baseline balance. Both the intubation and lithotomy success rates of ERAT reached 100%. No major complications occurred in either group postoperatively. ERAT was associated with a significantly shorter operative time (*p* = 0.035, *z* = −2.114) and shorter hospital stay (*p* = 0.002, *z* = −3.029) compared with LA. No recurrence of appendiceal fecalith was detected during the 3‐month follow‐up period.

(Figure 1: flow chart; Figure 2: ERAT operation for chronic fecalith appendicitis).

The overall median GSRS score across all patients was 4 (3) preoperatively and 2 (3) postoperatively, with a median improvement of 2 (2.5). In the ERAT group, the median preoperative GSRS score was 5 (4), which decreased to 1 (1.75) after surgery, yielding a median improvement of 3.5 (1). In the LA group, the median preoperative score was 4 (2), the postoperative score was 3 (3), and the median improvement was 1 (2). No significant between‐group difference was observed in preoperative GSRS scores. However, postoperative GSRS scores differed significantly between the two groups (*p* < 0.001, *z* = −3.512). Notably, the ERAT group achieved a substantially greater reduction in GSRS scores than the LA group (*p* < 0.001, *z* = −5.485).

### 3.2. Multivariate Analysis (Table 2)

**Table 2:** Multivariate analysis.

Characteristics	Odds ratio	95% confidence interval	*p*
Operative duration (min)	0.956	0.913–1.002	0.058
Hospital stay (days)	0.710	0.407–1.240	0.229
GSRS score (postoperation)	0.652	0.470–0.905	0.011
GSRS score (difference value)	2.137	1.461–3.126	**< 0.001**

*Note:* The bolded value indicates that statistical significance was defined as *p* < 0.05

Variables with statistical significance in univariate analysis, including operative duration, hospital stay, GSRS score (postoperation), and GSRS score (difference value), were entered into the multivariate regression model. Multivariate logistic regression demonstrated that the degree of GSRS score improvement was markedly higher in the ERAT group than in the LA group (*p* < 0.001, odds ratio = 2.137, 95% confidence interval: 1.461–3.126).

### 3.3. Postoperative Improvements of Each GSRS Subscale

Subgroup analysis of five GSRS domains revealed significant between‐group differences in postoperative improvements regarding abdominal pain, dyspepsia, and diarrhea. Nevertheless, no statistical differences were detected in the remission of reflux and constipation symptoms (Table 3).

**Table 3:** Postoperative improvements of each GSRS subscale.

Characteristics	Total	ERAT	LA	*p*	*t*/*z*
Abdominal pain symptoms score	1 (0)	1 (0)	1 (1)	**0.008**	−2.649
Reflux symptoms score	0 (1)	0 (1)	0 (1)	0.340	−0.954
Dyspepsia symptom score	0 (1)	1 (2)	0 (1)	**< 0.001**	−3.632
Constipation symptom score	0 (0)	0 (0.75)	0 (0)	0.099	−1.650
Diarrhea symptom score	0 (0)	0 (1)	0 (0)	**0.002**	−3.033

*Note:* The bolded value indicates that statistical significance was defined as *p* < 0.05

## 4. Discussion

Chronic appendicitis is an often‐overlooked condition that accounts for a large portion of abdominal pain cases and can lead to repeated medical consultations and exploratory surgery. Although acute appendicitis has typical clinical features, chronic appendicitis has atypical clinical features due to long‐term inflammation or fibrosis. Chronic appendicitis is characterized by intermittent abdominal pain, nausea, indigestion, and changes in bowel habits, which often makes it difficult to distinguish it from other gastrointestinal disorders, making diagnosis challenging [15]. Therefore, chronic appendicitis remains a topic of ongoing debate in the medical community. The role of fecalith obstruction in the appendix cavity is particularly noteworthy in chronic appendicitis, with studies suggesting that 10%–20% of appendicitis patients may have fecalith present [16]. The appendix is crucial not only for gastrointestinal function, but also for the physiological homeostasis of the whole body. Patients who undergo appendectomy may have an increased risk of Crohn′s disease, ulcerative colitis, *Clostridium difficile* infection, sepsis, and colorectal cancer [17].

This study revealed that ERAT produced more favorable outcomes in clinical symptoms and GSRS scores than LA. In patients with chronic fecalith appendicitis, ERAT markedly improves postoperative gastrointestinal function and relieves abdominal symptoms, and is thus a promising therapeutic modality. Notably, ERAT was superior to LA in alleviating abdominal pain, dyspepsia, and diarrhea. This discovery fills a critical literature gap and requires further investigation.

Abdominal symptoms of chronic fecalith appendicitis are commonly neglected in clinical practice, often resulting in misdiagnosis as functional bowel disorders such as functional dyspepsia, functional abdominal pain, and irritable bowel syndrome. Fecalith removal with ERAT can relieve patients′ symptoms and enable definitive diagnosis [9]. As an organ‐preserving endoscopic procedure, ERAT retains appendiceal function and protects gastrointestinal physiological function. Our findings offer meaningful clinical references of treatment strategies for chronic fecalith appendicitis.

Fecalith‐induced luminal obstruction of the appendix is the leading cause of abdominal pain throughout the disease course in patients with chronic appendicitis [18, 19]. The main abdominal pain sites were the right lower abdomen and periumbilical region. Previous studies have indicated that chronic abdominal pain following open appendectomy may be associated with surgical incisions and recurrent intestinal obstruction. However, long‐term follow‐up data of LA revealed a low incidence of such pain [20]. The inferior relief of chronic abdominal pain after LA compared with ERAT may be attributed to recurrent functional abdominal pain postoperatively, and the underlying mechanisms remain to be further explored. Patients with chronic appendicitis frequently suffer from recurrent abdominal pain and gastrointestinal dysfunction. ERAT can effectively alleviate these symptoms, improve patients′ quality of life and reduce procedure‐related risks [9]. Recurrent unexplained right lower quadrant pain has multiple etiologies, among which chronic appendicitis is one. Definite diagnosis is often challenging based on laboratory and imaging examinations [21]. Beyond therapeutic effects on chronic fecalith appendicitis, ERAT also offers diagnostic advantages. As a minimally invasive approach with good patient tolerance, it shows promising clinical value for appendicitis management. Importantly, the clinical implications of ERAT extend far beyond symptomatic relief; it also helps preserve and sustain normal gastrointestinal function.

The appendix is abundant in lymphoid tissue and serves a vital role in systemic immune regulation. It is also recognized as a reservoir for commensal bacteria, helping sustain the homeostasis of intestinal microbiota [22]. Studies have shown that chronic diarrhea is associated with bacterial dysregulation. Most of the patients with chronic diarrhea had changes in the dominant intestinal flora. The dominant bacteria such as anaerobic bacteria and G‐bacteria decreased significantly, whereas aerobic bacteria increased significantly. The number of enterobacterium increased, whereas *Lactobacillus* decreased significantly. *Enterococcus*, *Bifidobacterium*, and bacterioid bacteria showed a decreasing trend [23]. The appendix harbors diverse microorganisms, which can replenish healthy colonic microbiota after diarrheal episodes [24]. Accordingly, preserving and restoring appendiceal function via ERAT helps relieve dyspepsia and diarrhea, as well as associated symptoms including borborygmus, abdominal distension, and belching. *Clostridioides difficile* infection develops secondary to colonic flora imbalance. Previous research has demonstrated that appendectomy may exacerbate the severity of this infection, and early fecal microbiota transplantation represents a promising intervention [25]. Furthermore, studies have shown that appendectomy inhibits the activity of T‐helper 17 cells, inhibits autophagy, regulates molecules related to interferon activity, and inhibits immunopathology/vascular remodeling mediated by endothelin vascular activity [26]. As a key site for immunoglobulin A production, the appendix is essential for maintaining the quantity and function of intestinal flora. Given the bidirectional interaction between intestinal microecology and the gut immune system, preserving appendiceal function with ERAT contributes to the recovery of overall gastrointestinal function. Further studies are warranted to explore the dynamic alterations in intestinal microbiota following ERAT versus LA.

No significant differences were observed in the richness and diversity of luminal bacteria among patients with chronic diarrhea, whereas those of mucosal bacteria decreased markedly. Unlike luminal bacteria, colonic mucosal flora is also closely linked to chronic constipation [27]. In our study, the ERAT group achieved superior relief of diarrhea symptoms compared with the LA group, whereas no intergroup difference was found in the improvement of constipation. The gut microbiota and its metabolites (such as bile acids, short‐chain fatty acids, vitamins, amino acids, serotonin, and hypoxanthine) have been identified as possible etiological factors for irritable bowel syndrome. The abundances and metabolites of diarrheal and constipated microorganisms were different [28]. Accordingly, further in‐depth research is needed to compare the dynamic alterations in mucosal flora and their metabolites following ERAT and LA.

However, several limitations exist in the present study. Its retrospective design may compromise the generalizability of our findings. Large‐scale prospective multicenter trials are therefore required to further verify the sustained improvements in gastrointestinal function following ERAT versus LA for chronic fecalith appendicitis. Additionally, gastrointestinal function and symptomatic relief were evaluated using subjective scales, which are susceptible to patients′ subjective perceptions and reporting bias. Subsequent research should overcome these drawbacks to fully validate the clinical advantages of ERAT across diverse patient cohorts and different follow‐up periods.

## 5. Conclusion

In conclusion, ERAT serves as a viable alternative to LA for restoring gastrointestinal function and alleviating abdominal symptoms in patients with chronic fecalith appendicitis. Although this study is limited by its retrospective design, our findings merit further prospective research to validate the results and explore the potential mechanisms. Additionally, the present findings may facilitate the refinement of surgical techniques and optimization of nursing protocols for this patient population. They also support rational allocation of surgical resources and highlight the importance of individualized treatment strategies that take both short‐ and long‐term postoperative outcomes into account.

## Author Contributions

Yao Wu and Peichen Xia substantially contributed to the conception and design of the study. Jing Chen, Yao Liu, and Yanyan Wang performed the research and prepared the figures for the study. Yanyan Wang analyzed and interpreted the data and drafted the manuscript. Yan Yang and Wenzhuo Jiang critically revised the important intellectual content of the manuscript and provided final approval. Xiaomeng Jiang and Hong Zhu supervised the study. Yao Wu and Peichen Xia contributed equally to this work and share first authorship. Xiaomeng Jiang and Hong Zhu contributed equally to this work and share the corresponding authorship.

## Funding

This study was supported by the Key project fund of Jiangsu Provincial Health Commission (ZD2023016).

## Disclosure

All authors have read and approved the final manuscript.

## Ethics Statement

This study was performed in line with the principles of the Declaration of Helsinki. Approval was granted by the Ethics Committee of the Sir Run Run Hospital Affiliated to Nanjing Medical University (Approval No. 2022‐SR‐003).

## Conflicts of Interest

The authors declare no conflicts of interest.

## Data Availability

The data that support the findings of this study are available from the corresponding authors upon reasonable request.
